# Nonlinear modulation of COVID‐19 transmission by climate conditions

**DOI:** 10.1002/met.1985

**Published:** 2021-03-23

**Authors:** Meng Gao, Qiming Zhou, Xian Yang, Qingxiang Li, Shiqing Zhang, Ken Kin Lam Yung, Yike Guo

**Affiliations:** ^1^ Department of Geography, Faculty of Social Sciences Hong Kong Baptist University Hong Kong SAR China; ^2^ Department of Computer Science, Faculty of Science Hong Kong Baptist University Hong Kong SAR China; ^3^ School of Atmospheric Sciences Sun Yat Sen University Guangzhou China; ^4^ Department of Biology, Faculty of Science Hong Kong Baptist University Hong Kong SAR China

**Keywords:** COVID‐19, relative humidity, temperature, transmission

## Abstract

COVID‐19 is spreading rapidly worldwide, posing great threats to public health and economy. This study aims to examine how the transmission of COVID‐19 is modulated by climate conditions, which is of great importance for better understanding of the seasonal feature of COVID‐19. Constrained by the accurate observations we can make, the basic reproduction numbers (*R*_0_) for each country were inferred and linked to temperature and relative humidity (RH) with statistical analysis. Using *R*_0_ as the measure of COVID‐19 transmission potential, we find stronger transmission of COVID‐19 under mildly warm (0°C < *T* < 20°C) and humid (RH > 60%) climate conditions, while extremely low (*T* < −2°C) and high (*T* > 20°C) temperature or a dry climate (RH < 60%) weakens transmission. The established nonlinear relationships between COVID‐19 transmission and climate conditions suggest that seasonal climate variability may affect the spread and severity of COVID‐19 infection, and temperate coastal regions with mildly warm and humid climate would be susceptible to large‐scale outbreaks.

## Graphical abstract

The distribution of *R*_0_ with temperature (left) and relative humidity (right).

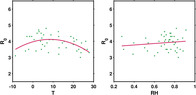

## 1 INTRODUCTION

A severe respiratory disease, identified later as the novel coronavirus disease 2019 (COVID‐19) caused by SARS‐CoV‐2 virus, was reported in Wuhan, Hubei province, China, in December 2019 (Wu *et al*., 2020). Since then, it has quickly spread around the world. As of June 29, 2020, over 10 million infections have been confirmed worldwide and over 500,000 people have died from the infection (JHU, 2020). COVID‐19 is characterized with notable ability to spread given that there is no effective vaccine and treatment to date.

The transmission of COVID‐19 is thought to occur through inhalation of droplets containing virus or through touching infected surfaces and then being infected via mouth, eyes or nose (Qu *et al*., 2020). The survival of virus in droplets and on infected surfaces or other medium can be largely affected by environmental conditions. An environmental study of SARS‐CoV‐1 found reduced survival of the virus at higher temperature and humidity (Chan *et al*., 2011). Several human coronaviruses (HCoV‐HKU1, HCoV‐OC43 etc.) also exhibit strong winter seasonality and become undetectable in summer months in temperate regions (Gaunt *et al*., 2010). Given the similarity between SARS‐CoV‐1 and other coronaviruses (SARS‐CoV‐2, HCoV‐HKU1, HCoV‐OC43 etc.), the spread of COVID‐19 might also be influenced by climate conditions.

Several environmental studies have attempted to link the transmission potential of COVID‐19 to temperature (*T*), relative humidity (RH) or other meteorological variables (Auler *et al*., 2020; Oliveiros *et al*., 2020; Tosepu *et al*., 2020; Wang *et al*., 2021). In epidemiology, the basic reproduction number (*R*_0_) and effective reproduction number (*R*_*t*_) are usually used to measure the transmission potential of a disease and how infectious a disease is in the real world. *R*_0_ denotes the expected number of cases generated by one case in a population, and *R*_*t*_ means the infectiousness at time *t*. When there is no intervention to control an outbreak, *R*_*t*_ would be equal to *R*_0_. Most previous studies used the daily number of confirmed infection cases or deaths to represent the transmission potential. For example, using the doubling time of the number of confirmed cases as a criterion of the rate of COVID‐19 spread, Oliveiros *et al*. (2020) concluded that higher temperature and lower humidity would lower the transmission of COVID‐19. Other studies assumed a serial interval of influenza or other diseases to obtain *R*_*t*_, and used it to measure the severity of infectiousness. For example, Wang *et al*. (2021) constructed *R*_*t*_ from an assumed serial interval of influenza and the daily number of infections in China, and they reported that high temperature and high humidity significantly reduce the transmission of COVID‐19.

Due to the high fraction of infections not detected by the health system and differences in testing policies, different proportions of infections were detected and reported across time and countries. The inappropriate selection of transmission potential would have led to questionable conclusions on the role of climate conditions. In addition, most of these studies adopted data for only one country or a limited number of countries. A limited span of climatic regimes considered would have also led to uncertainties in the conclusions.

The reported daily number of deaths is likely to be more reliable than the number of infections, and it is affected by only limited factors such as the quality of healthcare in different countries (Flaxman *et al*., 2020). This study aims to examine how climate conditions would affect the transmission, with *R*_0_ inferred from a more reliable methodology as the measure of transmission potential of COVID‐19.

## 2 DATA AND METHODOLOGY

### 2.1 *R*_0_ in different countries

We obtained the inferred *R*_0_ values from a recent study by Nouvellet *et al*. (2021). The daily death count data as of April 26, 2020, were taken from the European Centre for Disease Prevention and Control. Mobility information was sourced from Apple and Google, and the results inferred from Google mobility data are used in this study. For the Google data, six data streams, namely “residential,” “grocery and pharmacy,” “parks,” “transit stations,” “workplaces” and “retail and recreation,” are reported. The relative daily mobility for each country is quantified relative to the maximum mobility measured across the time series. The instantaneous reproduction number *R*_*t*_ was assumed as a function of relative mobility, and the delay between infection and deaths was also considered. More detailed descriptions of the estimation of *R*_0_ and *R*_*t*_ are documented in Nouvellet *et al*. (2021).

### 2.2 Climate datasets

We obtained hourly temperature and RH across the globe from the European Centre for Medium‐Range Weather Forecasts (ECMWF) ERA5 reanalysis dataset. The ERA5 reanalysis dataset was produced using the 4D‐Var data assimilation technique (Hersbach, *et al*., 2020). It provides climate variables on regular latitude–longitude grids at 0.25° × 0.25° resolution. The daily mean was calculated from extracted hourly temperature and RH in January, February and March for selected countries.

## 3 RESULTS AND DISCUSSION

### 3.1 Inferred *R*_0_ for selected countries

**FIGURE 1:**
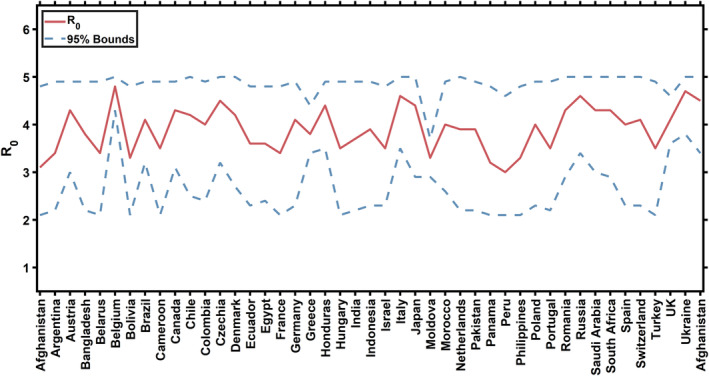
Inferred *R*_0_ with 95% confidence intervals for 45 countries

With observed reported deaths and mobility information, the basic reproduction numbers *R*_0_ for different countries were inferred. The effective reproduction number *R*_*t*_ has been demonstrated to be declining rapidly with implemented interventions (indicated also with mobility) in different countries, including school closure, city lockdown etc (Flaxman *et al*., 2020; Nouvellet 2021). Figure 1 displays the inferred *R*_0_ values with 95% confidence intervals for 45 countries across the globe. Only countries which fulfil the following three criteria were considered: (a) at least 10 deaths reported in the last week of data, (b) at least 10 deaths in the preceding week and (c) at least 100 deaths in total (Flaxman *et al*., 2020; Nouvellet 2021). As large‐scale intervention policies were implemented in China at an early stage of transmission and the reported cases are not suitable to represent the transmission of COVID‐19, we do not consider China in this study. Distinct differences are found in inferred *R*_0_ for different countries (Figure 1). Mid‐latitude countries, including the United Kingdom, Ukraine, Belgium, Italy etc., exhibit larger transmission potential (*R*_0_ > 4.5). Nordic countries present relatively lower transmission than other European countries (Figure 1). The inferred *R*_0_ values for countries in desert regions (Pakistan, Morocco and Egypt) are relatively smaller. Countries in tropical regions (Indonesia, Panama, Peru and Honduras) exhibit weaker transmission potential. The distribution of inferred *R*_0_ with region indicates that the transmission potential might be modulated by climate conditions.

### 3.2 Association between *R*_0_ and climate conditions

**FIGURE 2:**
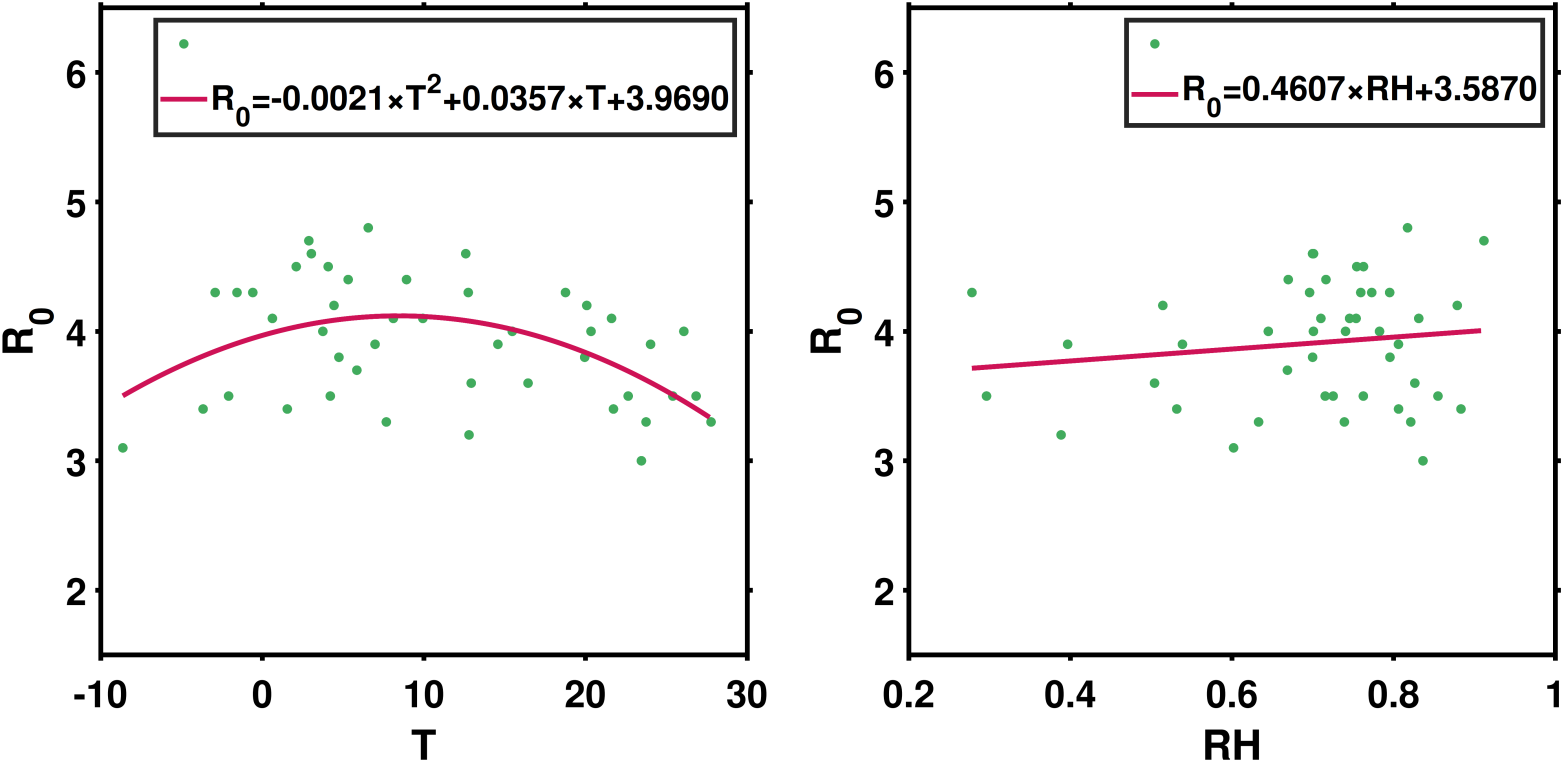
The distribution of *R*_0_ with temperature (left) and relative humidity (RH) (right)

In the estimation of transmission, *R*_*t*_ is affected primarily by intervention policies, and *R*_0_ is equal to *R*_*t*_ in the first several days after an outbreak (before interventions were implemented). As weather variables exhibit strong daily variations, we use 5 day averaged weather data to represent the climate conditions in each country. The first day was set as the beginning of transmission for each country. As displayed in Figure 2, *R*_0_ exhibits a unimodal distribution with temperature. At both higher temperature (*T* > 20°C) and lower temperature (*T* < −2°C), values of *R*_0_ exhibit relatively lower values (*R*_0_ < 3.5). Strong transmission (*R*_0_ > 3.5) occurs in areas with mildly warm climate (0°C < *T* < 20°C). We also observe low *R*_0_ values in regions with mildly warm climate and suspect that it might be modulated by RH. The distribution of *R*_0_ with RH suggests a weaker transmission under dry conditions (RH < 60%). Although climate in the desert regions are mildly warm, the dry conditions might result in lower transmission. We examined also other functions, but the current results can better represent the association (Table 1).

**TABLE 1:** *R* ^2^ and root mean square error of different fitting lines as functions of *T* and RH

Polynomial degree	1	2	3	4
*T*				
*R* ^2^	0.05	0.19	0.20	0.20
RMSE	0.46	0.42	0.42	0.42
RH				
*R* ^2^	0.02	0.02	0.04	0.05
RMSE	0.46	0.47	0.47	0.47

**FIGURE 3:**
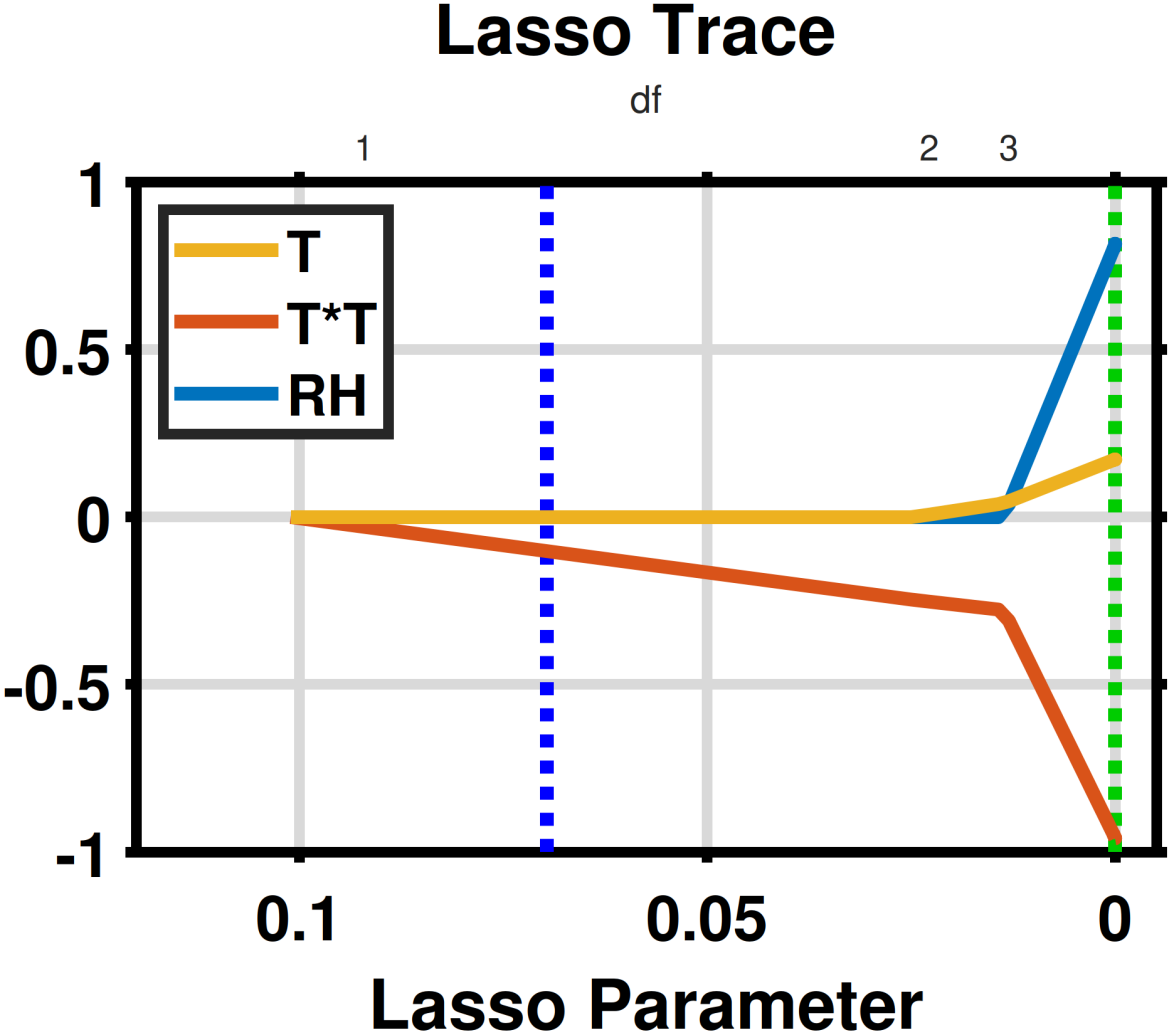
LASSO trace plots for *T*, square of *T* and relative humidity (RH)

To understand the relative significance of temperature and RH for the transmission of COVID‐19, we applied the least absolute shrinkage and selection operator (LASSO). LASSO was proposed by Tibshirani (1996), and it is featured both in feature selection and model interpretability. LASSO excels over other feature selection methods, such as stepwise selection, with a consideration of a penalty term which can shrink the regression coefficients toward zero to prevent overfitting. As Figure 2 shows that the values of *R*_0_ are related to the quadratic term of *T*, we compared the evolution of coefficients for *T*, *T* ^2^ and RH with the penalty term. When the penalty shrinks from negative to zero, the coefficient for the *T* ^2^ term first decreases (Figure 3), indicating the significance of the *T* ^2^ term.

We constructed a statistical model to describe the variations of inferred *R*_0_ in different countries, involving quadratic terms of *T* and linear terms of RH:

(1) $$R_{0}=aT^{2}+\mathrm{bT}+c\mathrm{RH}+d$$

**FIGURE 4:**
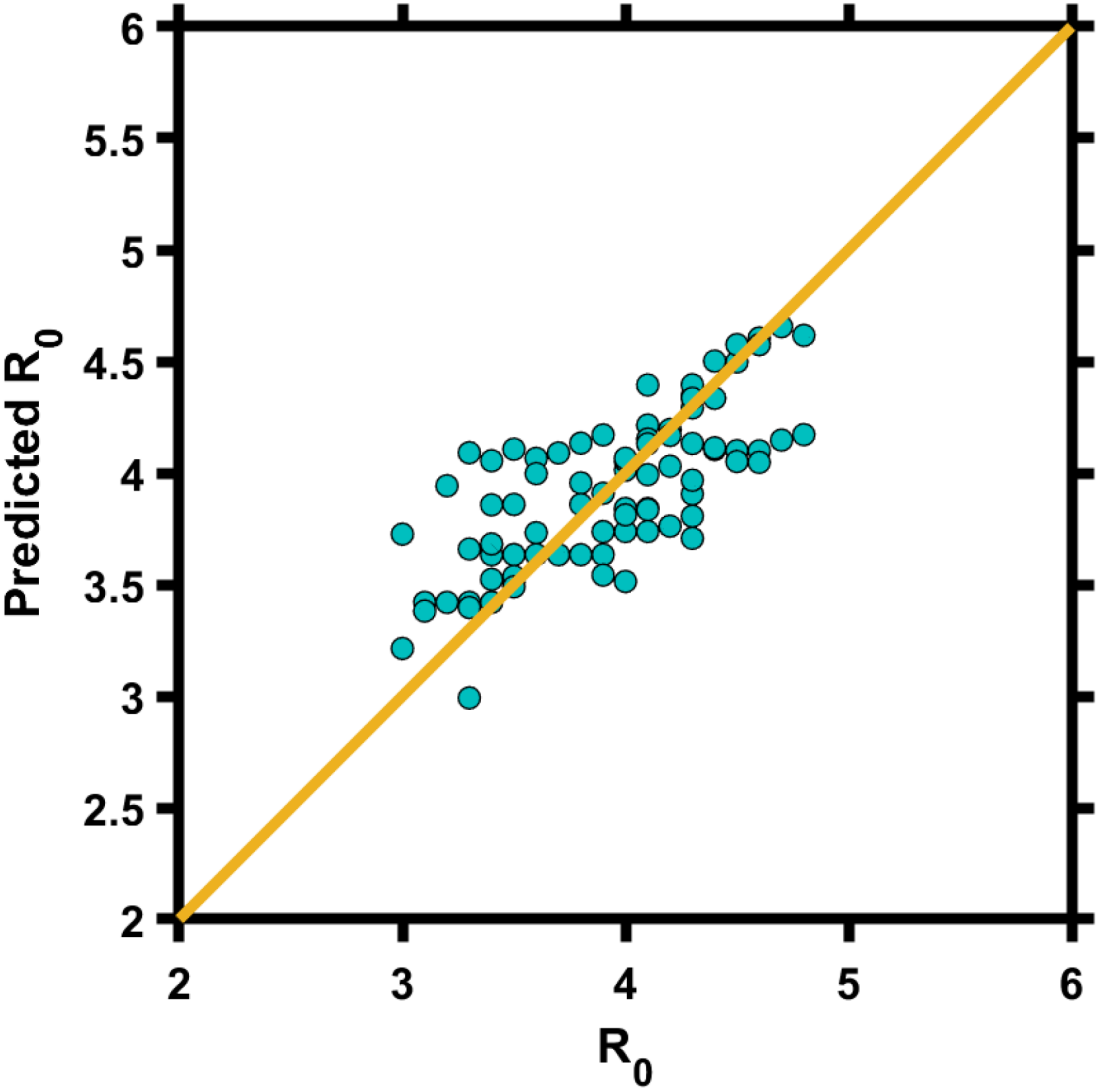
Scatter plots of predicted *R*_0_ with constructed statistical model and inferred *R*_0_ with transmission model

The scatter plot (Figure 4) of predicted *R*_0_ with inferred *R*_0_ with daily number deaths suggests a significant correlation (*r* = 0.50, *p* < 0.01). These results suggest that the transmission potential in different countries can be well represented with varied climate conditions across the globe.

## 4 DISCUSSION

Through back‐calculation of *R*_0_ constrained with daily number of deaths in countries with relatively good healthcare, we find stronger transmission of COVID‐19 under mildly warm (0°C < *T* < 20°C) and humid (RH > 60%) climate conditions, while extremely low (*T* < −2°C) and high (*T* > 20°C) temperature or dry climate (RH < 60%) weakens transmission. However, other studies claim different responses of COVID‐19 transmission to climate conditions. Wang *et al*. (2021) concluded that high temperature and high humidity would lower the transmission of COVID‐19. Oliveiros *et al*. (2020) reported also that high temperature would lower the transmission, but they found higher RH would favour the transmission. This finding of a relationship with RH is different from Wang *et al*. (2021) but consistent with ours. In addition to the questionable proxy used to represent transmission in these two studies, only data in China were considered. The span of climate conditions in China is limited, and the relationships found might not be applicable to extremely cold and dry areas, such as the Middle Eastern areas and Nordic countries.

Recent observations in two Wuhan hospitals (Liu *et al*., 2020) indicate that SARS‐CoV‐2 has the potential to be transmitted via aerosols. The growth of aerosols is largely affected by RH (Gao *et al*., 2020), and it was mentioned that higher virus survival would occur in droplets at high humidity levels. In summertime, the high temperature and wet removal of aerosols by monsoonal precipitation would be likely to suppress the transmission of COVID‐19. The nonlinear association between COVID‐19 transmission and temperature is mainly driven by data in Nordic countries, where complex interplays between temperature and human social behaviour could happen. The established relationships between COVID‐19 transmission and climate conditions suggest that seasonal climate variability may affect the spread and severity of COVID‐19 infection, and temperate coastal regions with mildly warm and humid climate would be susceptible to large‐scale outbreaks.

It should be kept in mind also that these findings should not be overclaimed. Climate conditions can only explain a limited amount of the variation in *R*_0_, and the remaining may be related to other factors, including population density, transportation etc. Besides, in the adopted methodology, although based on current best understanding of COVID‐19 in China, the distribution of the serial interval was assumed to be the same for the studied countries. This assumption might lead to uncertainties in the inferred *R*_0_ values, and the results can be further improved in future studies.

## CONFLICT OF INTEREST

We declare no conflict of interest.

## AUTHOR CONTRIBUTIONS

Concept and design: M.G., Y.G. Acquisition, analysis and interpretation of data: M.G., X.Y., Q.L., S.Z., K.K.Y., Y.G. Drafting of the manuscript: M.G., Y.G.

## ETHICS STATEMENT

This research did not involve human subjects. Obtaining institutional review board approval was not required.
